# Mapping of food environment policies in Zambia: a qualitative document analysis

**DOI:** 10.1186/s40795-023-00766-1

**Published:** 2023-10-02

**Authors:** Mulenga Mary Mukanu, Anne Marie Thow, Peter Delobelle, Zandile June-Rose Mchiza

**Affiliations:** 1 School of Public Health, University of the Western Cape, Bellville, 7535 South Africa; 2 Menzies Centre for Health Policy and Economics, University of Sydney, Camperdown, NSW 2006 Australia; 3 Chronic Disease Initiative for Africa, University of Cape Town, Cape Town, 7700 South Africa; 4 Department of Public Health, Vrije Universiteit Brussel, Brussels, Belgium; 5 Non-Communicable Diseases Research Unit, South African Medical Research Council, Cape Town, 7505 South Africa

**Keywords:** Food environments, Zambia, Healthy diets, Qualitative document analysis

## Abstract

**Background:**

The food environment in which people exercise food choices significantly impacts their dietary patterns. Policies that limit the availability, affordability, and access to unhealthy food while increasing that of healthier alternatives help build healthy food environments, which are required to address the double burden of malnutrition. This study aimed to assess the availability of food environment policies in Zambia.

**Method:**

We applied a two-step qualitative document analysis to identify policy content relating to healthy food environments from global and Zambia-specific nutrition-related policy documents. In the first step, global policy documents were analyzed to develop a reference point for globally recommended policies for healthy food environments. In the second step, Zambia’s nutrition-related policies were analyzed to identify content relating to healthy food environments. The identified policy content was then mapped against the global reference point to identify food environment policy gaps.

**Results:**

Our analysis of global policy recommendations identified five broad categories of policy provisions: information and education based; regulatory and legislative tools; strategies to promote production and access to healthy food production; social protection-based strategies and guiding principles for governments relating to multisectoral collaboration and governance. Our analysis found that Zambian Government policy documents in the health, agriculture, education, and national planning and development sectors have policy provisions for healthy food environments. While these policy provisions generally covered all five reference categories, we found policy gaps in the regulatory and legislative tools category relative to global recommendations.

**Conclusion:**

Zambia’s food environment policy landscape must include globally recommended regulatory and legislative policy measures like restricting the marketing of unhealthy foods and non-alcoholic beverages to children. Nutrition policy reforms are required to facilitate the introduction of regulatory and legislative policy measures that effectively address the double burden of malnutrition in Zambia.

## Introduction

Nutrition-related health problems continue to rank among the top causes of morbidity and mortality in many developing countries [1]. For instance, the 2021 *Global Nutrition Report* noted that 149.0 million children under five are stunted and 49.5 million are wasted, while 40.1 million are overweight and 677.6 million are obese. These health consequences of malnutrition have been estimated to cost the global economy up to $3.5 trillion per year [2].

The food environment in which people exercise food choices greatly impacts their dietary patterns. Food environments have been defined as ‘the physical, the economic, political and socio-cultural context in which consumers engage with the food system to make their decisions about acquiring, preparing and consuming food’ [3]. Food environment approaches to addressing nutrition problems challenge the dominant perspective that places the responsibility of good nutrition on individuals and neglects the role of environmental and structural factors, which are usually outside the health sector [4, 5]. For instance, regulatory policy interventions can help counter pervasive marketing strategies by unhealthy food industry which contributes to the obesogenic diets in many low and medium income countries [6–8].

Policy measures targeted at improving nutrition outcomes using a food environment approach aim to influence physical and economic access, promotion, advertising and information, and quality and safety of food [3]. These policy measures include social welfare initiatives, taxation of unhealthy foods, provisioning healthy foods, or limiting the marketing of unhealthy food to children [9–11]. Developing food environment policies therefore requires cross-sector coordination that facilitates the implementation of nutrition interventions in non-health sectors [12–15]. However, in many developing countries, improving nutrition outcomes often remains consigned to governments departments responsible for health, and to a lesser extent agriculture, with weak inter-sector coordination across these two government sectors [16]. As such, nutrition-related investments and policy solutions are often directed towards improving health care and the production of staple cereals to improve food security [17]. Few countries have successfully implemented the wide range of cross-sectoral policies that promote healthy food environments [9–11].

Zambia is currently experiencing a double burden of malnutrition where undernutrition (wasting, stunting, underweight), inadequate vitamins or minerals, overweight, obesity, and resulting diet-related noncommunicable diseases are present in the same population [18–20]. However, this double burden has emerged relatively recently, and little is known about how the Zambian government is positioned at policy level to address it. Zambia’s nutrition-related policy agenda for a long time focused on addressing the historically dominant problem of food insecurity, hunger and micronutrient deficiencies [21, 22]. More recently, political priority has increased for multisectoral policy intervention to reduce stunting, demonstrating the influence of emergent global policy priorities on the domestic nutrition policy agenda (ibid). Political science scholars suggest policy legacies (which include the performance of previous policies) are strong influencers of policy priorities of policymakers [23], and this has also been seen in a nutrition policy context [17]. Since governments use policies to formally communicate intent on an issue, one way to gain insight into the policy agenda and priorities of a government is to analyze current content of national policy documents. We applied a document analysis methodology to assess the availability of nutrition-related policies for healthy food environments in Zambia’s national policies and assessed them against international recommendations to identify gaps. A mapping of Zambia’s current nutrition policy landscape is a good starting point for strengthening and reforming nutrition policy as it will help document any existing gaps and identify priority areas for investment.

## Methods

A qualitative document analysis was applied in this study. Document analysis is a research method that primarily uses written documents to systematically investigate a phenomenon of interest. The process of document analysis generally involves the identification, selection, retrieval and interpretation of both physical and electronic documents to develop new empirical knowledge [24]. While there are many approaches to implementing a document analysis, we applied Dalglish et al.’s [25] ‘READ’ approach in this study because it provided a replicable systematic approach.

### Conceptual framework

We used the High Level Panel of Experts on Food Security and Nutrition (HLPE)’s definition of food environments [3] to conceptualize food environments and to develop our inclusion criteria for the study. The HLPE is the science-policy interface of the Committee on World Food Security, which is, at the global level, the foremost inclusive and evidence-based international and intergovernmental platform for food security and nutrition. In 2017, the HLPE produced a report that analyzed the association between food systems people’s dietary patterns and nutritional status The report also included recommendation of actions required to transform food systems into support healthy and sustainable diets [3].

According to the HLPE, physical and economic access to food (proximity and affordability); food promotion, advertising and information; and food quality and safety are the key elements of the food environment that influence food choices, food acceptability and diets [3]. This study only focused on policies for physical access, economic access and promotion and marketing of food as described in Table 1.

**Table 1:** Inclusion criteria and search terms

Physical access
This dimension relates to the physical availability of healthy food in close proximity to consumers. Examples include policies that encourage healthier diets through public procurement of foods and policies and investments that support diversification and the production of nutrient-rich foods (e.g. fruits and vegetables and legumes)
Key search terms: healthy diet, healthy food, healthy food access, food security
Economic access
This dimension relates to influence of affordability and the price of healthy food. Policy measures under this component might involve making nutritious foods cheaper and unhealthy foods more expensive. Examples include discriminatory trade policies and taxations of unhealthy food options or subsidies on healthy food options like fruits and vegetables
Key search terms: unhealthy food tax, healthy food subsidies, healthy food affordability, trade policy
Promotion, advertising and information
This dimension relates to exposure of consumers to information that will influence their food choices. This might involve limiting the exposure of vulnerable populations such as children to industry driven promotions of unhealthy foods or conducting public health campaigns that promote healthy food habits. Examples include education-based interventions to improve public awareness of healthy dietary behaviours; strengthening regulations for advertising and marketing and increasing transparency of information on labels
Key search terms: Food marketing to children, unhealthy food advertisements to children, healthy food choices

### Identification of documents

This document analysis was centered on two types of policy documents: global policy documents with nutrition-related recommendations and Zambia’s government policy documents. Documents were identified using the criteria presented in Table 1. This study only considered policy documents published between 2002 and 2021. We chose 2002 as a reference point because it was the year WHO released the World Health Report [26] which drew global attention to actions required to promote healthy lives by reducing diet related risk factors among others.

Global policy documents were identified from websites of institutions and organizations that issue nutrition related policy recommendations, including the World Health Organization (WHO), the Food and Agriculture Organization (FAO) and the United Nations (UN) and its affiliated organizations of which Zambia is a member state. Documents of interest included global strategic plans, frameworks of action, and global action plans. Search terms used included those based on the food environment domains (see inclusion criteria) and document type key words *‘global strategic plan’, ‘global strategy’, ‘global policy’, ‘global standards’, global guidelines’, ‘framework of action’, global recommendations’,* and *‘global action plan’.* The search term was built by combining the food environment related and document type key words e.g. ‘*healthy diet strategic plan’* or ‘*global standards unhealthy food advertisements’*. The document search was primarily conducted on Google search engine and in the search bar on the global organization’s main website or on the publications or resources pages of the website. Additional documents were identified by checking through the references of the initial documents.

Zambia’s policy documents were identified by checking through the publication section on the websites of government ministries and organizations, including the Ministries of Health, Education, Agriculture, Youth, Sports and Child Development, Community Development and Social Services, Commerce Trade, and Industry and National Development Plan for their policy documents. Previous research shows that these government ministries have nutrition-relevant mandates [22]. In the case where a policy that meets the inclusion criteria was expired and had not yet been renewed, we included the expired version in the review. For policies that are reviewed periodically, like the national development plans, we included the latest version in the review. In order to capture possible policies that might not have been available on the online platform searched, we validated the list of policies identified with officials from the education, health and agriculture sectors where the majority of nutrition policies are concentrated as well as the National Food and Nutrition Commission.

A total of eleven global and eighteen Zambian policy documents were included in the analysis as shown in Table 2.

**Table 2:** Global policy recommendations for healthy food environments

**Category I: Strategies to promote food and nutrition education and awareness of healthy diets**
1. Include nutrition (health literacy) in the curricula offered in primary and secondary schools [27–30]
2. Develop national food or nutrient based guidelines [30]
3. Implement mass media/public and social marketing campaigns based on national dietary guidelines to promote healthy diets and consumption of nutritious foods including traditional foods [28, 31, 32]
**Category II: Regulations and legislative tools**
4. Implement tax measures on unhealthy foods [28–30, 32]
5. Provide incentives such as subsidies for production of healthy food options including reformulation [28–30, 32, 33]
6. Implement comprehensive policy on labeling of food [28–30, 32]
7. Regulate marketing of food and non-alcoholic beverages to children including in schools [29, 30, 32–35]
**Category III: Strategies to promote healthy food production and access to healthy food**
8. Strengthening of agriculture policy to improve supply of locally grown nutritious food [27–30, 33]
9. Strengthen linkages between production, demand and consumption of nutritious food through value chain development [27, 33]
10. Adopt policies that support healthy diets at school by engaging food retailers and caterers including school tuck-shops to improve the availability, affordability and acceptability of healthier food products [32, 33] and limit the availability of products high in salt, sugar and fats [29]
11. Explore potential of urban and peri-urban agriculture initiatives such as school and community gardens and issuing contracts to local food growers to supply fresh produce and support the diversification of school meals and diets of schoolchildren and adolescents in cities [27, 29]
12. Develop and enforce national food safety legislation and regulations ensure that food producers and suppliers throughout the food chain operate within internationally recognized standards, guidelines and codes of practice on food safety and quality [28–30]
**Category IV: Social protection strategies**
13. Incorporate nutrition objectives into social protection measures for vulnerable populations e.g. cash transfer and school feeding program [28, 34]
**Category V: Guiding principles for governments**
14. Governance • Provide political will and commitment to nutrition [28, 30] • Coordinate action and ensure policy coherence across sectors such as agriculture, youth, recreation, sports, education, commerce and industry, finance, transportation, media and communication, social affairs and environmental and urban planning [28–30, 34, 35] • Establish nutrition coordinating mechanism such as task force or advisory bodies [29, 34] • Develop responsive, culturally appropriate policies to the specific country context [27, 28, 32, 35] • Provide means and platform for monitoring progress towards targets [28, 30, 36]
15. Multisectoral collaboration • Engage all relevant stakeholders including NGOs, civil society, communities, the private sector and the media pupils, parents [27, 29, 30, 36] • Manage potential conflicts of interests [35, 36] • Integrate nutrition objectives into food and agriculture policy, programme design and implementation, to enhance nutrition sensitive agriculture, ensure food security and enable healthy diets [30, 36]

### Data analysis

The focus of our analysis was to identify policy content relating to healthy food environments from global and Zambia-specific nutrition-related policy documents and was conducted in two steps. The first step of the document analysis was used to develop a ‘reference point’ or analytical framework of globally recommended policies required for healthy food environments. In the second stage, Zambia’s nutrition-related policies were analyzed to identify content relating to healthy food environments. The identified policy provisions were then mapped against the reference point developed in Stage 1 to identify food environment policy gaps.

#### Step 1: Developing a reference point (analytical framework) of global policy recommendations for healthy food environments

Our analysis followed the READ approach for document analysis as proposed by Dalglish et al. 2020 [25]: (1) ready materials, (2) extract data, (3) analyze data and (4) distil your findings.

Firstly, the identified policy documents were screened to assess the relevance to nutrition and food environments. Secondly, a more detailed review of the content was conducted to identify data to be extracted. We extracted content from the documents relating to the three dimensions of food environments i.e. physical access, economic access and promotion and marketing of food (see Table 1). The extracted excerpts together with information on the year of publication, issuing institution and objective were transferred to an excel spreadsheet for analysis. Thirdly, thematic analysis was used to make meaning of the extracted data by identifying subthemes under each of the three dimensions of the food environments used in the inclusion criteria. The thematic analysis is a suitable approach of identifying, describing, and interpreting key patterns within and across cases of interest. In the fourth and final step, themes and patterns identified from the extracted data were distilled into a list of global healthy food environment policy recommendations (Table 2).

#### Step 2: Mapping Zambia’s nutrition policies against global policy recommendations for healthy food environments

The READ approach as described above was used. However, in the fourth step, framework analysis was used to map data from the Zambia policy documents against the analytical framework of global policy recommendations developed in Step 1. Framework analysis is a form of thematic which is useful for conducting comparative cross-sectional analysis of qualitative data using a combination of data description and abstraction and mainly consists creating an analytic framework and applying this analytic framework [37]. Using the framework analysis approach, we were able to identify the policy gaps in Zambia’s policy provisions relative to the global recommendations. This approach of identifying policy gaps has been used in other nutrition-related studies [38–41].

Trustworthiness of the analysis was assured in multiple ways. Firstly, a senior government official from the National Food and Nutrition Commission checked the Zambian policy documents identified and verified that they covered all the available government policies relevant to nutrition in Zambia. Secondly, the co-authors of the paper verified the relevance of data extracted from the documents to the study and the appropriateness of the subsequent themes developed. Thirdly, the findings from the analysis were presented and validated by twelve nutrition stakeholders from the government, UN agencies and NGOs through a stakeholder consultative meeting.

## Results

### Global policy recommendations for healthy food environments

Table 2 shows the list of global recommended policy measures for healthy food environments based on the global policy documents analyzed in this study (see Table 3). Using thematic analysis, we categorized the policy recommendations into four main groups. The education-related policy recommendations under Category I were oriented toward improving knowledge and consequently improving the capacity of people to make healthy food choices. Category II captured the regulatory and legislative policy recommendations with the potential to impact the availability, affordability, accessibility, and marketing of food in the food environment. Measures for improving access to healthy food through increase production of nutritious food were captured in Category III. The policy recommendations in Category IV focused on using social protection structures to provide nutrition-related interventions especially targeted at vulnerable populations. Category V included general guiding principles that governments can use when implementing nutrition policy such as governance structures and multisectoral collaboration. Our analysis found strong coherence in food environment policy measures recommended by the different global institutions across the five categories.

**Table 3:** List of policy documents analyzed

Document title	Year	Organization/institution
**Global policy documents**		
1. Global Strategy for Diet and Physical Activity	2004	World Health Organization
2. Set of recommendations on the marketing of foods and non-alcoholic beverages to children	2010	World Health Organization
3. Comprehensive Implementation Plan for Maternal, Infant and Young Child Nutrition	2014	World Health Organization
4. Global Action Plan for the Prevention and Control of Non-Communicable Diseases (NCDs) 2013–2020 (extended to 2030)	2014	World Health Organization
5. Second International Conference on Nutrition: Framework of Action	2014	World Health Organization and Food and Agriculture Organization
6. Sustainable Development Goals (SDGs)	2016	United Nations
7. UN Decade of Action on Nutrition 2016 to 2025	2017	United Nations
8. Global Action Plan on Physical Activity 2018–2030	2018	World Health Organization
9. UN Political Declaration on NCDs	2018	United Nations
10. Legal guide on school food and nutrition: legislating for a healthy school food environment	2020	Food and Agriculture Organization
11. Voluntary Guidelines on Food Systems and Nutrition	2021	Food and Agriculture Organization
**Zambia’s policy documents**		
1. Vision 2030	2005 – 2030	Ministry of Finance
2. The Competition and Consumer Protection Act	2010	Government of the Republic of Zambia
3. Food Safety Act	2019	Government of the Republic of Zambia
4. Food and Nutrition Act	2020	Government of the Republic of Zambia
5. Seventh National Development Plan	2017 – 2021	Ministry of Planning and National Development
6. Educating Our Future: National Education Policy	2006	Ministry of Education
7. National School Health and Nutrition Policy	2008	Ministry of Education
8. Guidelines for implementing School Health Nutrition Programme activities	2008	Ministry of Education
9. National Food and Nutrition Policy	2006	National Food and Nutrition Commission
10. National Food and Nutrition Strategic plan	2017	National Food and Nutrition Commission
11. Most Critical Days Programme II: “Zambia's Five Year Flagship Stunting Reduction Programme”	2018 – 2020	National Food and Nutrition Commission
12. National Health Policy	2012	Ministry of Health
13. Adolescent Health Strategic plan	2012	Ministry of Health
14. Zambian Strategic Plan 2013–2016 Non-Communicable Diseases and Their Risk Factors	2013	Ministry of Health
15. National Social protection Policy	2014	Ministry of Community Development, Mother and Child Health
16. National Health Strategic Plan 2017 – 2021	2017	Ministry of Health
17. National Youth Policy	2015	Ministry of Youth, Sport and Child Development
18. National Agriculture Policy	2016	Ministry of Agriculture

### Mapping Zambia’s policy provisions for healthy food environments against global recommendations

Table 4 shows the mapping of food environment related content extracted from Zambian Government policy documents against the global recommendations for healthy food environments. Our analysis found that Zambia has policies provisions that support healthy food environments under all the five categories. These policy recommendations were mainly found in policy documents from the health, education, agriculture, and national planning and development sectors. The policy provisions were categorized as ‘explicit’ and ‘general’ based on the author’s judgement of how well the policies fit the inclusion criteria (See Table 1). The explicit category included policies that directly addressed the main dimensions of the food environments that the study was focusing. The general category included policy provisions that were judged to be broad and whose primarily focus was not food environment.

**Table 4:** Mapping of Zambian policy provisions related to the food environment against global policy recommendations

Global policy recommendation	Zambian policy recommendations	
	Explicit policy provision	General policy provisions
**Category I: Strategies to promote food and nutrition education and awareness of healthy diets**		
1. Include nutrition (health literacy) in the curricula offered in primary and secondary schools [27–30]	Schools shall ensure institutionalized health and nutrition education and initiate nutrition clubs in schools and communities [42] to promote consumption of healthy food, educate learners and the community on how to read and understand food labels and symbols and how to make good food choices when buying [43, 44] and disseminate dietary guidelines and nutrition education and counseling materials [44]	
2. Develop national food or nutrient based guidelines [30]	Zambia developed food-based guidelines [45]	
3. Implement mass media/public and social marketing campaigns based on national dietary guidelines to promote healthy diets and consumption of nutritious foods including traditional foods [28, 31, 32]	Promote population health education programmes to raise awareness of NCDs and their risk factors, to empower individuals, families and communities with appropriate knowledge to develop and practice healthy lifestyles and behavior change and improve the health status of adolescents [42, 46–51] Encourage consumption of nutrient rich food including traditional foods such as edible insects and caterpillars to improve dietary diversity [46, 52]	
**Category II: Regulatory and legislative tools**		
4. Implement tax measures on unhealthy foods [28–30, 32]	Advocate for public policies that support and promote health by reforming the legal and regulatory framework for food and nutrition to support healthy environments, good nutrition and prevent NCDs risk factors [47, 48]	
5. Provide incentives such as subsidies for production of healthy food options including reformulation [28–30, 32, 33])		
6. Implement comprehensive policy on labeling of food [28–30, 32]	Promote mandatory nutrition labeling for all prepackaged foods [52]	Prohibition of deception in labeling, packaging and selling of food. A person shall not label, package, sell or advertise any food in a manner that is false, misleading or deceptive as regards its character, nature, value, substance, quality, composition, merit or safety or in contravention of this Act [53] A product that is sold in Zambia shall have a label to clearly indicate the product name, the ingredients used in the product, the date of manufacture and expiry of the product, the manufacturer’s name, the physical location of the manufacturer, the telephone number and any other contact details of the manufacturer [54]
7. Regulate marketing of food and non-alcoholic beverages to children including in schools [29, 30, 32–35]		Prohibition of sale or advertisement of food as treatment for diseases: A person shall not—(a) advertise any food as treatment, preventative or cure for a disease, disorder or abnormality; or (b) sell any food that is presented on the label or is advertised as a treatment, preventative or cure for a disease, disorder or abnormality [53]
**Category III: Strategies to promote production of and access to healthy food**		
8. Strengthening of agriculture policy to improve supply of locally grown nutritious food [27–30, 33]	Develop and/or advocate for policies and programmes that will ensure food and nutrition security, food quality and safety at individual household, community and national level [46, 55] Government shall promote cultivation and consumption of indigenous crop varieties [46, 55]	
9. Strengthen linkages between production, demand and consumption of nutritious food through value chain development [27, 33]	Enhance agriculture value chains through investment in production, agro-processing and marketing, including export market and distribution mechanisms. Value chain development will promote the participation of small and medium enterprises, coupled with provision of business development services to enterprises along different value chains. Emphasis will be placed on promotion of citizen participation in agri-business and linking local to regional and international value chains [47]	
10. Adopt policies that support healthy diets at school by engaging food retailers and caterers including school tuck-shops to improve the availability, affordability and acceptability of healthier food products [32, 33] and limit the availability of products high in salt, sugar and fats [29]	To improve feeding and eating practices, schools shall: 1) establish a tuck shop which should be selling healthy foods 2) encourage the family to prepare and pack healthy foods for leaners 3) ensure that learners are counseled on good feeding practices 4) encourage consumption of traditional food [43] Promote the provision and availability of healthy foods in all public and private institutions including schools, other education institutions and work places [52] Government shall ensure that food production units are revitalized in all learning institutions; and are used to teach children improved food production methods and agro entrepreneurship skills [42]	
11. Explore potential of urban and peri-urban agriculture initiatives such as school and community gardens and issuing contracts to local food growers to supply fresh produce and support the diversification of school meals and diets of schoolchildren and adolescents in cities [27, 29]	Improve Dietary Diversification through Nutrition-Sensitive Agriculture by increasing production and consumption of dietary-diverse nutrient-dense foods by 1) Develop and scale up a comprehensive homestead food production model 2) Support school demonstration gardens in production of micronutrient rich foods [44]	Develop urban and peri-urban economies with a priority on development and implementation of Integrated Development Plans. The policy focus will be on promoting entrepreneurship, creating urban industrial clusters, strengthening value-chain linkages and improving infrastructure in formal settlements [47]
12. Develop and enforce national food safety legislation and regulations ensure that food producers and suppliers throughout the food chain operate within internationally recognized standards, guidelines and codes of practice on food safety and quality [28–30]	Government enacted the Food Safety Act of 2020 which contains provisions for the production, manufacture, handling, preparation and storage of food in a manner that prevents food related diseases and harm [53] Implement programmes aimed at promoting maintenance of a clean, healthy environment and good nutrition including food safety regulation to ensure safe drinking water and sanitary conditions [47]	
**Category IV: Social protection strategies**		
13. Incorporate nutrition objectives into social protection measures for vulnerable populations e.g. cash transfer and school feeding program [28, 34]	Enhance food security and nutrition through programmes such as supplementary and school feeding enhancement and nutritious foods and household food security promotion through improved targeting and coverage of social protection programs like social cash transfers [42, 43, 47, 56]	
**Category V: Guiding principles for governments**		
14. Governance • Provide political will and commitment to nutrition [28, 30] • Coordinate action and ensure policy coherence across sectors such as agriculture, youth, recreation, sports, education, commerce and industry, finance, transportation, media and communication, social affairs and environmental and urban planning [28–30, 34, 35] • Establish nutrition coordinating mechanism such as task force or advisory bodies [29, 34] • Develop responsive, culturally appropriate policies to the specific country context [27, 28, 32, 35] • Provide means and platform for monitoring progress towards targets [28, 30, 36]	Government should establish a national NCD coordinating committee (or equivalent) with membership by all ministries and a national multi-sectorial NCD Action Plan, with full participation of non-health ministries and non-state actors. Government should strengthen leadership for enforcing existing legislation and regulations [57]	Government should enhance leadership and governance for the social determinants and risk factors for NCDs by establishing and maintaining an efficient institutional arrangement and strong nutritional networks to strengthen nutrition programming [48, 49, 57] Government will facilitate the ratification and domestication of all international and regional agreements, conventions, declarations and protocols on health to which the country is a signatory to [48]
15. Multisectoral collaboration • Engage all relevant stakeholders including NGOs, civil society, communities, the private sector and the media pupils, parents [27, 29, 30, 36] • Manage potential conflicts of interests [35, 36] • Integrate nutrition objectives into food and agriculture policy, programme design and implementation, to enhance nutrition sensitive agriculture, ensure food security and enable healthy diets [30, 36]	The second phase of the Most Critical Days Program (MCDP) will, therefore, continue to advance the multi-sector approach initiated under the first phase in order to encourage simultaneous roll out and implementation of the high impact nutrition interventions by the line ministries and the range of stakeholders involved in the MCDPII implementation [44] Ministry of Education will cooperate closely with agencies that work to improve the nutritional, health, sanitary and environmental health status of communities to which the school pupils belong to [42] Promote awareness among Government employees and the community at large that, health problems can only be adequately solved through multi-sectoral collaboration involving such sectors as Education, Agriculture, Water, Private Sector, including not for profit and faith based organisations [48]	

Information and education-based policy recommendations of Category I were available in policies from the education and health sectors. The government through the Ministry of Education (MoE) has incorporated nutrition education into the national school curriculum in order to improve the nutrition knowledge and dietary habits of learners. In addition, the government through the Ministry of Health (MoH) and its agencies like the National Food and Nutrition Commission (NFNC) has plans for population-based health awareness campaigns that will address nutrition-related risk factors to health. These awareness campaigns will be anchored in the national food-based dietary guidelines which were recently launched in 2021 through the Ministry of Agriculture with support from other nutrition-related sectors. The overall focus of the education is to improve the health of learners.

Our analysis found policy gaps under the regulatory and legislative policy recommendations of Category II. None of the policy documents analyzed included policies or plans to implement taxation on unhealthy food or provide incentives for the production of healthy food options. Policies supporting the regulation of marketing of food and non-alcoholic beverages to children including in school environments were also absent in the documents included in this study. Policy provisions for food labeling were however available. The government through the Ministry of Health in the NCD strategic plan of 2013 to 2016 did plan for mandatory nutrition labeling for all prepackaged foods, but this plan has since expired. More recent legislation generally addresses labeling and advertisements of all food. In the Competition and Consumer Protection Act of 2010, the Government prohibits deception in the labeling, packaging, and selling of food. While the Act has provisions that stipulate the information to be included on a food label, information on the dietary quality of the food or its health implications is not a requirement. We found no policy provisions for front of pack labelling system, a key requirement for fostering healthy consumer food choices.

Policy strategies for promoting the production of and access to healthy food under Category III were mainly found in the Ministry of Agriculture and Education policy documents. The key agriculture-related policies under this category include the promotion of cultivation and consumption of diverse indigenous foods through nutrition-sensitive agriculture and investments in agriculture value chains. The education-related policies promote access to healthy foods in learning institutions by ensuring vendors in the school sell healthy foods among other provisions. The government also plans to revitalize food production in learning institutions to further enhance access to healthy food by learners and surrounding communities. In addition, food safety is regulated through the Food Safety Act which is a domestication of the Codex Alimentarius [58] and has provisions relating to food standard food items produced or imported and sold to consumers.

The government’s policy recommendation under Category IV of social protection strategies are aimed at enhancing food and nutrition security for vulnerable populations. Policy provisions largely focused on making social protection interventions including the Public Welfare Assistance Scheme, Social Cash Transfer Scheme, Nutrition and Supplementary Feeding interventions such as the Home-Grown School Feeding Programme more nutrition sensitive. For instance, the government plans to link social cash transfers with the promotion of appropriate feeding and care practices and provision of micro-nutrients [56]. Nutrition sensitive social protective policy provision were found primarily in the whole of government, education and agriculture sector documents.

The guiding principles of Category V found in the governance and coordination mechanisms section which was included in the majority of policy documents. In these sections, the government called for multisectoral collaboration for implementation of government policies. A few documents such as school health nutrition policy [42] of the education sector and the national health policy (Ministry of Health, 2012) in the health sector explicitly included a list of stakeholders and their roles in implementation while the rest broadly called for collaboration among key stakeholders including government ministry, non-governmental organizations, private sector and beneficiaries.

Evidence of policy coherence across some government sectors was present. Policy documents from health, education and agriculture sectors all had provisions that aims to encourage/promote consumption of healthy food in the population [43–45]. Homestead food production models that promote production of diverse, nutrient dense foods was a priority in both health [44] and agriculture [46] sector policy documents. Nutrition sensitive social protection measures were present in whole of government [47] as well as education [43], health [48] and agriculture sector policy documents [46]. The government’s nutrition related social protection programs include school feeding programs, social cash transfers and food security packs whose aim is to improve access to nutritious food by vulnerable populations.

## Discussion

The current study aimed to assess the availability of nutrition related policies for healthy food environments in Zambia and to identify existing policy gaps, using global recommendations as a reference point. Our analysis of global policy recommendations identified five broad categories of policy provisions: information and education based; regulatory and legislative tools; strategies to promote production and access to healthy food production; social protection-based strategies and guiding principles for governments relating to multisectoral collaboration and governance. Our analysis found that government policy documents in the health, agriculture, education and national planning and development sectors have policy provisions for healthy food environments. While these policy provisions generally covered all the categories of the framework, we found policy gaps in the regulatory and legislative tools category relative to global recommendations.

Government regulatory and legislative policies are critical for building healthy food environments as they limit availability, affordability and access of unhealthy food while increasing that of healthier alternatives [59, 60]. The most commonly used regulatory and legislative policy interventions are targeted at limiting the amount of harmful ingredients like sugar, salt and fats in foods. While the cost effectiveness and public health impact of these interventions have been demonstrated [61], it is widely acknowledged that they are difficult to develop and implement mainly due to perceived negative impact on economic sectors [62]. For instance, South Africa is one of the few African country that has managed to implement a ‘sugar tax’ at 10–11% per litre of sugar sweetened drinks for health purposes [63]. In contrast, the small tax in place in Zambia was introduced primarily for revenue raising purposes [22, 64]. This weak regulatory and legislative policy environment for nutrition in Zambia is likely to be exploited by the ‘big food’ industry as they look to expand to newer markets in developing countries [65, 66].

Policy development theories show that successful prioritization and effective policy design are not only dependent on government and political will. The key stages in policy development of complex problems like nutrition, including agenda setting, policy formulation and implementation, require a combination of actors and are therefore prone to actor influences according to their interests and power [67, 68]. Evidence shows that activities of industry affiliated actors like lobbying and litigation against governments continues to challenge the introduction of effective measures, such as taxations, in many countries [69–74]. In Zambia, lobbying by industry and government stakeholders from the economic sector was instrumental in the very minimal sugar tax of 3% being recommended [22]. Adding to the complexity of regulatory and legislative food environment policy measures is the fact that implementation requires additional policy frameworks outside the primary sector. Our analysis identified key regulatory frameworks such as the competition and consumer protection act, food safety act and food and nutrition act that can be leveraged to introduce and/or strengthen regulatory policies, including marketing restrictions and labeling. In addition, there is evidence of cross sectoral policy coherence on some nutrition goals in Zambia’s policy documents. There is a critical opportunity for the Government of Zambia to further strengthen the coherence of policies across key nutrition sensitive sectors to increase the likelihood of implementing cross sectoral programmes like taxations and ensure the policy measures in one sector do not undermine the goals of other sectors.

Although the Government of Zambia encourages multisectoral coordination for nutrition policy implementation in policy content—which is an important first step—achieving strong multisectoral cooperation is challenging. Evidence supports multisectoral approaches to addressing nutrition-related problems over traditional vertical programming as the latter has little success in effectively addressing the multifaceted drivers of the double burden of malnutrition [75, 76]. Key lessons for successful integration from countries like Ethiopia include strong political will with national prioritization of nutrition coupled with strong governance structures that promote accountability among stakeholders [77]. In Zambia, multisectoral coordination is still suboptimal despite the availability of policy provision supporting multisectoral collaboration identified in this study and presence of governance structures such as the nutrition coordination committee at provincial and district level that have been reported in literature [78, 79]. Additional mechanisms for implementing and measuring multisectoral collaboration might be required to strengthen nutrition coordination. These might include incentives that will motivate stakeholders to pursue collaboration as most policy actors weigh the perceived costs and benefits of collaborating [80], identification of a champion and development of a strategy that outlines the key responsibility for each stakeholder [81].

The major strength of the current study is that it demonstrated that qualitative document analysis is a useful tool for policy analysis that can be used to assess availability of policies and when coupled with an appropriate reference point (in this case, globally recommendations policy measures), can identify gaps in the policy environment. The policy gaps so identified can be the basis of additional research or advocacy activities. Our findings contribute to the evidence body on the status of food environment policies in developing countries that are facing the double burden of malnutrition.

However, the findings in this current paper are limited by our reliance on historical documentary data; the government’s policy priorities could have evolved since the publication of the documents. Policy availability in this study implied the presence of official documentation of government plans on a subject through the official channels of communication. However, government policies communicate aspiration or intent as availability of policy does not guarantee implementation. Therefore, to further the body of knowledge on healthy food environments in Zambia, future research should examine how the available policies identified in this study have been translated into programs and/or evaluate the impact of policies on nutrition related outcomes. For instance, the INFORMAS Food-EPI framework [82] can be used to rate the implementation of available food environment policies in Zambia. An analysis of the interests and power relations among nutrition policy actors would be useful to further understand the nutrition agenda setting in Zambia which is a key determinant of the choice of policies that are prioritized [83, 84].

## Conclusion

The nutrition policy landscape in Zambia includes some policy provisions that foster healthy food environments in the five categories identified from the review of global recommendations. These policies span government sectors including health, education and agriculture. However, the lack of recommended regulatory and legislative policies including taxation of unhealthy foods and restrictions on marketing of unhealthy food and non-alcoholic beverages to children is a key policy gap that might require policy reforms if the country is effectively address the double burden of malnutrition present in the population.

## Data Availability

All data generated or analysed during this study are included in this published article.

## Abbreviations

FAO: Food and Agriculture Organization
MoE: Ministry of Education
MoH: Ministry of Health
NCD: Non-communicable diseases
NFNC: National Food and Nutrition Commission
WHO: World Health Organization
UN: United Nations
